# High-Resolution Computed Tomography and Intraoperative Correlation in Cholesteatoma: Enhancing Preoperative Evaluation and Surgical Management

**DOI:** 10.7759/cureus.44333

**Published:** 2023-08-29

**Authors:** Abha A Kapoor, Abhay Kapoor, Nimisha U Nimkar, Hiren D Soni, Vishnu S Ojha, Ratnadeep Biswas

**Affiliations:** 1 Department of Otorhinolaryngology, Gujarat Medical Education and Research Society (GMERS) Medical College and Hospital, Gotri, Vadodara, IND; 2 Department of Internal Medicine, B.J. Medical College, Ahmedabad, Ahmedabad, IND; 3 Department of Internal Medicine, All India Institute of Medical Sciences, Patna, Patna, IND

**Keywords:** predictive value of tests, sensitivity and specificity, diagnostic test accuracy, intra-operative finding, otitis media, x-ray computed tomography, hrct, high-resolution computed tomography, middle ear cholesteatoma, cholesteatoma

## Abstract

Introduction: Cholesteatoma, a hazardous non-neoplastic lesion of the temporal bone, is prevalent in socio-economically disadvantaged groups in developing nations like India. Timely detection and surgical intervention are essential for effective management. High-resolution computed tomography (HRCT) has revolutionized the assessment of temporal bone pathology, though its role in preoperative evaluation remains debated. This study aimed to validate HRCT's utility in diagnosing cholesteatoma, compare its findings with intraoperative observations, and assess sensitivity and specificity.

Methods: This diagnostic accuracy study was conducted at a tertiary care center in Western India, from March 2021 to November 2022. HRCT findings of 54 cholesteatoma patients were evaluated and compared with intraoperative findings. Sensitivity, specificity, positive predictive value (PPV), negative predictive value (NPV), accuracy, and Cohen's kappa coefficient were calculated.

Results: HRCT demonstrated a sensitivity exceeding 90% in identifying scutum erosion, mastoid sclerosis, and abnormalities in the tympanic membrane, along with a specificity surpassing 90% in detecting various conditions, including facial canal erosion, sinus plate erosion, lateral semicircular canal erosion, erosion of the posterior wall of the external auditory canal, and abnormalities in the tympanic membrane. Furthermore, HRCT exhibited an accuracy of over 90% in detecting most pathologies. There was a perfect or near-perfect agreement observed for abnormal tympanic membrane, sinus plate erosion, mastoid sclerosis, and erosion of the posterior wall of the external auditory canal (with kappa values > 0.8). Moderate to fair agreement was noted for other pathologies.

Conclusion: HRCT offered precise detection of the majority of pathologies, thereby facilitating surgical planning. However, the presence of limitations in distinguishing specific abnormalities highlights the significance of utilizing HRCT in tandem with other diagnostic modalities to ensure meticulous diagnosis and effective treatment planning.

## Introduction

Cholesteatoma is a dangerous non-neoplastic lesion of the temporal bone, prevalent in socio-economically disadvantaged groups in developing nations like India [1]. It is characterized by the abnormal growth of keratinizing squamous epithelium, which possesses invasive properties leading to destructive changes in the middle ear cleft and potentially causing serious complications in both intracranial and extracranial regions [2].

Efficient management of cholesteatoma relies on timely detection and appropriate surgical intervention. The emergence of high-resolution computed tomography (HRCT) scanning has transformed the assessment of temporal bone pathology [2].

The role of HRCT in the preoperative evaluation of cholesteatoma remains a contentious topic. It aids in evaluating disease extension, surgical planning, and identification of potential complications, given its ability to provide excellent topographic visualization. Nevertheless, its capability to determine the extent of soft tissue involvement in the antrum, middle ear, and posterior tympanic spaces, is invaluable in guiding surgical decision-making. The precise evaluation of individual temporal bone anatomy, including any variations, along with the extent of disease, helps surgeons in selecting the appropriate surgical approach and minimizing complications [3].

Studies have evaluated the accuracy and usefulness of preoperative CT scans in detecting cholesteatoma and planning surgical management [4]. While some studies have reported limitations in differentiating cholesteatoma from other pathologies, HRCT is considered extremely helpful for assessing ear pathologies, especially for detecting osseous damage. HRCT's superior topographic visualization aids in devising appropriate surgical plans [5].

Thus, this study aimed to validate HRCT's utility in determining middle ear structures' status in cholesteatoma, assess its sensitivity and specificity in diagnosing the condition, and compare preoperative findings with intraoperative observations.

## Materials and methods

Study design and setting

This diagnostic accuracy study was conducted at the Department of Otorhinolaryngology (ENT), Gujarat Medical Education and Research Society (GMERS) Medical College and Hospital, Gotri, Vadodara, India, in collaboration with the Department of Radiology from March 2021 to November 2022.

Ethical considerations

The study received approval from the institutional human ethics committee, following the guidelines and principles outlined in the Declaration of Helsinki. Written informed consent was obtained from all participants before their inclusion in the study. Strict confidentiality and privacy were maintained throughout the study. The Institutional Human Ethics Committee of GMERS Medical College and Hospital, Gotri, (ECR/28/Inst./GJ/2013/RR-19) issued approval BHR/20/2021.

Study population

Patients diagnosed with cholesteatoma who underwent operative correction after evaluation with HRCT of the temporal bone were considered.

Inclusion and Exclusion Criteria

The study included patients of all ages and both sexes who presented with cholesteatoma and provided written informed consent. However, pregnant women, patients for whom CT was contraindicated, individuals considered unfit for surgery or anesthesia, those who did not undergo surgery after HRCT evaluation, patients with middle ear pathologies other than cholesteatoma and congenital ear diseases, as well as individuals with a history of previous ear surgery were excluded from the study.

Sampling technique

Complete enumeration; all eligible patients who presented to the ENT outpatient department (OPD) during the 18-month study period (March 2021-November 2022) and met the inclusion criteria were included in the study.

Study protocol

In this study, patients who had been clinically diagnosed with cholesteatoma at the ENT OPD were identified as potential participants. Each of these patients underwent a thorough evaluation, including a detailed ear examination and history-taking to understand the nature and duration of symptoms like discharge and hearing loss, as well as other complaints. Additionally, examinations of the nose and throat were conducted. The diagnosis of cholesteatoma and its stage were established based on factors such as patient history, otoscopic findings, and reports from post-operative histopathology of middle ear tissue obtained during surgery.

HRCT scans of the temporal bone were then carried out on all patients using a SOMATOM Emotion 16-slice CT scanner (Siemens AG, Erlangen, Germany) with 2 mm axial and coronal slices. The resulting HRCT images were meticulously evaluated by two experienced radiologists independently. Any differences in interpretation were resolved through discussion and consensus, with a third radiologist consulted if needed.

During the surgical procedures, careful observation and documentation of intraoperative findings were conducted for each patient. These findings were taken as the reference standard for comparison. The surgeries were conducted under strict sterile conditions, adhering to aseptic precautions, and with the use of general anesthesia.

A comprehensive comparative analysis was then performed, juxtaposing the findings from HRCT temporal bone scans with the observations made during surgery to assess the correlation between the radiological and surgical findings.

Statistical analysis

The data was collected and entered into Microsoft Excel (Microsoft, Redmond, WA, USA) and analyzed using IBM SPSS Statistics for Windows, Version 26.0. (IBM Corp., Armonk, NY, USA). Categorical variables were expressed as proportions. The sensitivity, specificity, positive predictive value (PPV), negative predictive value (NPV), and accuracy of HRCT (index test) in detecting various pathologies were calculated, with the intraoperative findings considered as the reference standard. Cohen's kappa coefficient (k) was also calculated to assess the agreement between the HRCT and intraoperative findings. The kappa values were interpreted as follows: values from 0 to 0.20 were considered as slight agreement, 0.21 to 0.40 as fair agreement, 0.41 to 0.60 as moderate agreement, 0.61 to 0.80 as substantial agreement, and 0.81 to 1 as almost perfect agreement.

## Results

A total of 54 patients were recruited in the study, with a mean (standard deviation) age of 32.35 (16.14) years, and 32 (59.3%) of them were females. Among the participants, 32 (59.3%) had infection of the left ear. The most common chief complaint was ear discharge, present in 31 (57.4%) participants, followed by the presence of both ear discharge and reduced hearing in 20 (37%) participants. A majority of 27 (50%) had conductive hearing loss, while 26 (48.1%) had a mixed type of hearing loss (Table 1). The clinical characteristics on otoscopy, pure tone audiometry, and intraoperative findings of the patients are given in Table 2.

**Table 1:** Demographic details of the patients (N=54)

Variable	Category	Counts (Percentage)
Age (years)	1-20	17 (31.5)
	21-40	19 (35.2)
	41-60	15 (27.8)
	≥61	3 (5.6)
Gender	Male	22 (40.7)
	Female	32 (59.3)
Ear infected	Right	22 (40.7)
	Left	32 (59.3)
Chief complaints	Ear discharge	31 (57.4)
	Reduced hearing	3 (5.6)
	Both	20 (37)
Type of hearing loss	Conductive	27 (50)
	Sensorineural	1 (1.9)
	Mixed	26 (48.1)

**Table 2:** Clinical findings in patients with cholesteatoma (N=54)

Evaluation	Structure	Category	Counts (Percentage)
Otoscopy	Tympanic membrane	Attic retraction pocket	16 (29.6)
		Posterosuperior quadrant retraction pocket	28 (51.9)
		Others	10 (18.5)
Pure tone audiometry	Right ear	No impairment	8 (14.8)
		Slight impairment	17 (31.5)
		Moderate impairment	24 (44.4)
		Severe impairment	5 (9.3)
		Profound impairment	0 (0)
	Left ear	No impairment	13 (24.1)
		Slight impairment	10 (18.5)
		Moderate impairment	23 (42.6)
		Severe impairment	6 (11.1)
		Profound impairment	2 (3.7)
Intraoperative	Scutum	Eroded	48 (88.9)
		Normal	6 (11.1)
	Malleus	Completely necrosed	12 (22.2)
		Partially necrosed	28 (51.9)
		Intact	14 (25.9)
	Incus	Completely necrosed	24 (44.4)
		Partially necrosed	25 (46.3)
		Intact	5 (9.3)
	Stapes	Completely necrosed	7 (13)
		Partially necrosed	27 (50)
		Intact	20 (37)
	Facial canal (vertical)	Eroded	3 (5.6)
		Normal	51 (94.4)
	Facial canal (horizontal)	Eroded	4 (7.4)
		Normal	50 (92.6)
	Sinus plate	Eroded	4 (7.4)
		Normal	50 (92.6)
	Mastoid	Sclerosed	39 (72.2)
		Pneumatized	15 (27.8)
	Lateral semicircular canal	Eroded	2 (3.7)
		Normal	52 (96.3)
	Posterior wall of external auditory canal	Eroded	7 (13)
		Normal	47 (87)
	Tympanic Membrane	Abnormal	51 (94.4)
		Normal	3 (5.6)

The sensitivity, specificity, PPV, NPV, and accuracy of HRCT in detecting various pathologies were calculated. It was observed that HRCT had a sensitivity of more than 90% in detecting scutum erosion, mastoid sclerosis, and abnormalities in the tympanic membrane. Additionally, HRCT demonstrated a specificity of more than 90% in detecting facial canal (vertical) erosion, facial canal (horizontal) erosion, sinus plate erosion, lateral semicircular canal (SCC) erosion, erosion of the posterior wall of external auditory canal, and abnormalities in the tympanic membrane. The overall accuracy of HRCT was above 90% in detecting almost all pathologies, except for erosion in the malleus, incus, and stapes.

It was observed that there was a perfect or near-perfect agreement between the HRCT and the intraoperative findings in detecting sinus plate erosion, mastoid sclerosis, erosion of the posterior wall of external auditory canal, and abnormalities in the tympanic membrane (kappa values > 0.8). Substantial agreement was found in detecting scutum erosion, malleus erosion, and facial canal (vertical) erosion (kappa value > 0.6), moderate agreement in detecting stapes erosion, facial canal (horizontal) erosion, and lateral SCC erosion (kappa value > 0.4), and fair agreement in detecting incus erosion, with a kappa value of 0.256 (Table 3).

**Table 3:** Diagnostic characteristics of HRCT in detecting various pathologies in patients with cholesteatoma (N=54) HRCT- High-resolution computed tomography, PPV- Positive predictive value, NPV- Negative predictive value

Pathology	HRCT Finding		Intraoperative Finding		Sensitivity (%)	Specificity (%)	PPV (%)	NPV (%)	Accuracy (%)	Kappa value
	Detected (n)	Not Detected (n)	Present (n)	Absent (n)						
Scutum erosion	47	7	48	6	95.8	83.3	97.9	71.4	94.4	0.738
Malleus erosion	38	16	40	14	87.5	78.6	92.1	68.8	85.2	0.631
Incus erosion	42	12	49	5	81.6	60	95.2	25	79.6	0.256
Stapes erosion	36	18	34	20	88.2	70	83.3	77.8	81.5	0.595
Facial canal (vertical) erosion	2	52	3	51	66.7	100	100	98.1	98.1	0.791
Facial canal (horizontal) erosion	4	50	4	50	50	96	50	96	92.6	0.460
Sinus plate erosion	3	51	4	50	75	100	100	98	98.1	0.847
Mastoid sclerosis	40	14	39	15	97.4	86.7	95	92.9	94.4	0.859
Lateral semicircular canal erosion	2	52	2	52	50	98.1	50	98.1	96.3	0.481
Erosion of the posterior wall of external auditory canal	6	48	7	47	85.7	100	100	97.9	98.1	0.913
Abnormal tympanic membrane	51	3	51	3	100	100	100	100	100	1

## Discussion

Patient history and otoscopic examination play a crucial role in diagnosing cholesteatoma. However, in cases where there is a presence or suspicion of cholesteatoma, HRCT is a supplementary examination in the preoperative evaluation [6].

The overall accuracy of HRCT was above 90% in detecting almost all pathologies, except for erosion in the malleus, incus, and stapes. A strong agreement was observed between the HRCT scans of the temporal bone and the surgical findings for various abnormalities. The kappa statistics indicated a perfect agreement (k = 1) for abnormal tympanic membrane, almost perfect agreement for sinus plate erosion (k = 0.847), mastoid sclerosis (k = 0.859), and erosion of the posterior wall of external auditory canal (k = 0.931). Furthermore, there was substantial agreement observed for scutum erosion (k = 0.738), malleus erosion (k = 0.631), and facial canal (vertical) erosion (k = 0.791). However, a moderate radio-surgical agreement was found for stapes erosion (k = 0.595), facial canal (horizontal) erosion (k = 0.460), and lateral SCC erosion (k = 0.481). A fair agreement was seen with incus erosion (k = 0.256).

This study included a total of 54 subjects, with 22 males and 32 females. The female predominance observed in this study is consistent with a study by Gamra et al. [7]. This disparity in gender distribution might be attributed to factors such as higher incidences of malnutrition and anemia in association with upper respiratory infections in rural areas, affecting females more.

X-ray has traditionally been utilized for the evaluation of middle ear pathologies, including cholesteatoma. However, the limitations of X-ray, such as poor soft tissue resolution and reduced sensitivity in detecting subtle bone erosions, have made it less favorable in modern clinical practice [8].

When compared to magnetic resonance imaging (MRI), HRCT has certain advantages in the evaluation of cholesteatoma. HRCT provides better spatial resolution for bony structures, making it particularly useful in detecting bone erosions and anatomical variations associated with cholesteatoma. This capability aids in surgical planning and decision-making, allowing surgeons to precisely identify the extent of disease and select appropriate surgical approaches [9].

Middle ear opacification can be caused by conditions such as chronic infection, glomus tympanicum, vascular anomalies, neoplasms, or hemotympanum. Unfortunately, due to its contrast resolution, HRCT lacks specificity in differentiating between these various causes [4,10]. Here, the role of MRI emerges due to its superior soft tissue contrast, making it more effective in evaluating soft tissue involvement and potential intracranial or extracranial extensions of cholesteatoma. MRI's ability to distinguish cholesteatoma from other soft tissue pathologies becomes valuable in cases where the diagnosis is not conclusively determined based on HRCT findings alone [9,11]. While our study centered on HRCT, it's important to acknowledge the complementary role of MRI in cases where intricate soft tissue characterization is essential. Incorporating advanced imaging modalities like diffusion-weighted (DW) MRI, as recommended by recent studies [12], could further enhance our understanding of cholesteatoma and its differentiation from other middle ear pathologies. However, given the limitations of our study, exploring these innovative approaches would require separate investigations in the future.

In our study we found a strong agreement between HRCT and intraoperative findings for sinus plate erosion, mastoid sclerosis, and scutum. A similar outcome has been reported in a study by Manik et al. [6]. However, they observed a weaker correlation for the facial nerve canal.

Additionally, we observed a notable discrepancy in the kappa value concerning facial canal erosion, indicating a stronger association in the vertical part of the facial canal compared to the horizontal part. We postulate that the relatively shorter and more tortuous nature of the horizontal facial canal, in contrast to the vertical part, gives rise to this anatomical complexity [13]. Consequently, this complexity can pose challenges in achieving accurate imaging and interpretation on HRCT. As a result, the visualization and precise identification of the horizontal segment may be comparatively difficult, leading to reduced concordance with intraoperative findings. However, this could also be attributed to the utilization of 2 mm cuts in HRCT as employed in this study.

According to Gaurano et al. [14], showing facial nerve canal involvement before surgery is frequently challenging, not only because of the facial nerve canal's small size but also due to its oblique orientation and the presence of developmental dehiscence, especially when adjacent to soft tissue.

In our study, we found a lower sensitivity and kappa value for incus erosion suggesting poor agreement in detecting incus erosion in the correlation between HRCT and intraoperative findings. This observation could be attributed to the advanced nature of the cases encountered in our setting. Patients referred to tertiary centers often present with more advanced and complex cholesteatoma cases, resulting in extensive soft tissue involvement near the ossicles. A previous study by Çakan et al. [15], reported that involvement of multiple ossicles reduced diagnostic sensitivity for cholesteatoma on HRCT, which is a possible explanation for the findings of the study. Another factor to take into consideration is the employment of 2 mm cuts in our study, which could have heightened the possibility of missing pathologies in smaller ossicles like incus and stapes. Opting for thinner cuts, such as 1 mm, might potentially improve the sensitivity in detecting erosion within these structures.

There are certain limitations to this study as well. Unfortunately, HRCT lacks specificity in differentiating soft tissue which should be investigated with MRI [6]. Furthermore, using thinner cuts than the 2 mm cuts employed in this study could potentially yield advantageous results for smaller structures such as incus and stapes. Despite efforts to reduce subjectivity in interpreting the HRCT scans, a certain level of dependency on the observers’ judgment remains.

## Conclusions

HRCT demonstrated over 90% accuracy in detecting most pathologies and generally showed good agreement with intraoperative findings. It offers detailed information about bony structures and essential anatomical landmarks, enabling surgeons to anticipate affected areas, select suitable surgical techniques, and reduce the risk of accidental injuries during mastoid surgery. However, while HRCT provides significant advantages in detecting middle ear diseases, it may not always offer a complete view of certain abnormalities. Like any medical tool, HRCT should be used alongside other diagnostic modalities to ensure precise diagnosis and make appropriate treatment decisions.
